# Neurally Adjusted Ventilatory Assist vs. Conventional Mechanical Ventilation in Adults and Children With Acute Respiratory Failure: A Systematic Review and Meta-Analysis

**DOI:** 10.3389/fmed.2022.814245

**Published:** 2022-02-22

**Authors:** Mengfan Wu, Xueyan Yuan, Ling Liu, Yi Yang

**Affiliations:** Jiangsu Provincial Key Laboratory of Critical Care Medicine, Department of Critical Care Medicine, Zhongda Hospital, School of Medicine, Southeast University, Nanjing, China

**Keywords:** neurally adjusted ventilatory assist, acute respiratory failure, asynchrony index, patient-ventilator asynchrony, conventional mechanical ventilation

## Abstract

**Background:**

Patient-ventilator asynchrony is a common problem in mechanical ventilation (MV), resulting in increased complications of MV. Despite there being some pieces of evidence for the efficacy of improving the synchronization of neurally adjusted ventilatory assist (NAVA), controversy over its physiological and clinical outcomes remain. Herein, we conducted a systematic review and meta-analysis to determine the relative impact of NAVA or conventional mechanical ventilation (CMV) modes on the important outcomes of adults and children with acute respiratory failure (ARF).

**Methods:**

Qualified studies were searched in PubMed, EMBASE, Medline, Web of Science, Cochrane Library, and additional quality evaluations up to October 5, 2021. The primary outcome was asynchrony index (AI); secondary outcomes contained the duration of MV, intensive care unit (ICU) mortality, the incidence rate of ventilator-associated pneumonia, pH, and Partial Pressure of Carbon Dioxide in Arterial Blood (PaCO2). A statistical heterogeneity for the outcomes was assessed using the *I*^2^ test. A data analysis of outcomes using odds ratio (OR) for ICU mortality and ventilator-associated pneumonia incidence and mean difference (MD) for AI, duration of MV, pH, and PaCO2, with 95% confidence interval (CI), was expressed.

**Results:**

Eighteen eligible studies (*n* = 926 patients) were eventually enrolled. For the primary outcome, NAVA may reduce the AI (MD = −18.31; 95% CI, −24.38 to −12.25; *p* < 0.001). For the secondary outcomes, the duration of MV in the NAVA mode was 2.64 days lower than other CMVs (MD = −2.64; 95% CI, −4.88 to −0.41; *P* = 0.02), and NAVA may decrease the ICU mortality (OR =0.60; 95% CI, 0.42 to 0.86; *P* = 0.006). There was no statistically significant difference in the incidence of ventilator-associated pneumonia, pH, and PaCO2 between NAVA and other MV modes.

**Conclusions:**

Our study suggests that NAVA ameliorates the synchronization of patient-ventilator and improves the important clinical outcomes of patients with ARF compared with CMV modes.

## Introduction

Mechanical ventilation is regarded as an effective method and is widely used in the treatment of critically ill patients with acute respiratory failure (ARF) to maintain adequate gas exchanges (1). However, with traditional modes of mechanical ventilation (MV), the mismatching between the demand of patient and the level of assistance may produce a patient-ventilator asynchrony and leads to poor clinical outcomes, such as increased airway pressure, delayed triggering, and excessively loaded respiratory muscles, which can give rise to respiratory fatigue, asynchrony index (AI) increasing, and, eventually, extend the duration of MV (2–4). Consequently, optimizing the strategies for improving the synchronization of patient-ventilator has been a crucial goal to reduce adverse clinical complications and outcomes.

Neurally adjusted ventilatory assist (NAVA) is a ventilation mode, which controls the time and intensity of ventilation assistance through the electrical activity of the diaphragm (EAdi) (1). Different from the CMV mode, mechanical breath is triggered by the patient's inspiratory effort and enables the patient to influence the machine-cycling to a varying extent (5). In previous studies, NAVA is associated with a better patient-ventilator interaction, both in adult and in pediatric patients (6, 7). However, the controversy of the differential impacts of NAVA on physiologic and clinical outcomes remains. Furthermore, large randomized controlled trials (RCTs) are needed to clarify whether these potential physiologic benefits may improve the clinical prognosis (8).

This study aims to assess the effects of NAVA on the patient-ventilator interaction and clinical outcomes in patients with ARF compared with CMV modes.

## Methods

This systematic review and meta-analysis adhere to the applicable Preferred Reporting Items for Systematic Reviews and Meta-Analyses (PRISMA) guidelines.

### Eligibility Criteria

We included all randomized controlled trials (RCTs) and randomized crossover trials. Studies were eligible if they (i) compared NAVA with the conventional mechanical ventilation mode in patients with ARF, (ii) included outcomes such as AI or secondary outcomes, (iii) were published in English. We did not include trials from neonates, especially premature infants, as this is completely another population and respiratory distress syndrome (RDS) in infants is a different pathology compared with acute respiratory distress syndrome (ARDS) in adults and children.

Asynchronies were classified into six types: (a) ineffective triggering (missed effort); (b) ineffective inspiratory triggering; (c) double-triggering; (d) auto-triggering; (e) a prolonged cycle; and (f) a short cycle (9). The AI, defined as the number of asynchrony events divided by the total respiratory cycles computed as the sum of the number of ventilator cycles (triggered or not) and of wasted efforts (2, 9), was the primary outcome. The secondary outcomes included the duration of MV, ICU mortality, and the incidence rate of ventilator-associated pneumonia.

### Search Strategy

An ordinary database retrieval of PubMed, EMBASE, Web of Science, Medline, Cochrane Central Register of Controlled Trials, trial registers, and gray literature from 2008 to October 2021 was executed. The articles of those published were restricted to English. Sea terms included “NAVA,” “neurally adjusted ventilatory assist,” “ARF,” and “acute respiratory failure. In PubMed, we used a neurally adjusted ventilated assist” or “NAVA,” and “ARF” or “acute respiratory failure” for search strategy. Furthermore, the retrieved literature contained the bibliographies of all relevant studies and reviews to confirm the potentially qualified studies.

### Selection of Studies

The search results were merged, and the duplicate records of the same report were removed. Two authors (MF and XY) have independently sifted all study titles and abstracts to determine the initial search strategy for potential eligibility and retrieved the potentially related studies for a full-text review.

### Assessment of Risk of Bias

The risk of bias of the involved trials included in this meta-analysis was assessed according to the recommendations of the Cochrane Handbook of Systematic Reviews of Interventions in the following domains: selection bias (a random sequence generation and allocation concealment), performance bias (blinding of participants and personnel), detection bias (blinding of outcome assessment), attrition bias (incomplete outcome data), and reporting bias (selective outcome reporting) (http://handbook.cochrane.org). Jadad scale was used to calculate the quality of every enrolled study. The quality appraisal was mostly based on whether the authors added quality appraisal indicators (e.g., whether the article showed the concealment of randomization, whether it showed the randomization number occurring) in their articles.

### Statistical Analysis

All statistical analyses were accomplished with Review Manager 5.3 [The Nordic Cochrane Centre, The Cochrane Collaboration (28)] and StataSE12.0. Data analysis of the continuous outcome was expressed as mean difference (MD) with 95% CI, while data analysis of the dichotomous outcome was expressed as odds ratio (OR) with 95% CI. To statistically aggregate the data from the included studies, we used the method proposed by Liu et al. (29) to convert the median along with the 25 and 75% percentiles to mean and standard deviation. Statistical heterogeneity for the outcomes was assessed using the *I*^2^-test. We considered *I*^2^ greater than or equal to 50% and a *p*-value of less than 0.1 as high heterogeneity (30). Funnel plots and Egger's test were used to evaluate the publication bias on the primary outcome (31). The choice of fixed-effect and random-effect models depended on statistical heterogeneity. If it is *p* < 0.10 or *I*^2^ > 50%, we used a random effect to combine data; otherwise, the fixed-effect model was chosen. Meta-regression was used to explore the source of heterogeneity. Meanwhile, we used a sensitivity analysis to evaluate the robustness and the reliability of the combined results. Forest plots were generated to demonstrate the individual study data, as well as the pooled data for each endpoint. For the primary outcome, subgroup analyses were performed to compare AI grouped by age (i.e., adult, pediatric), ventilation methods [i.e., invasive ventilation, non-invasive ventilation (NIV)], and the cause of ARF (i.e., COPD, others) because of the high heterogeneity.

## Results

### Study Characteristics

**Figure 1:**
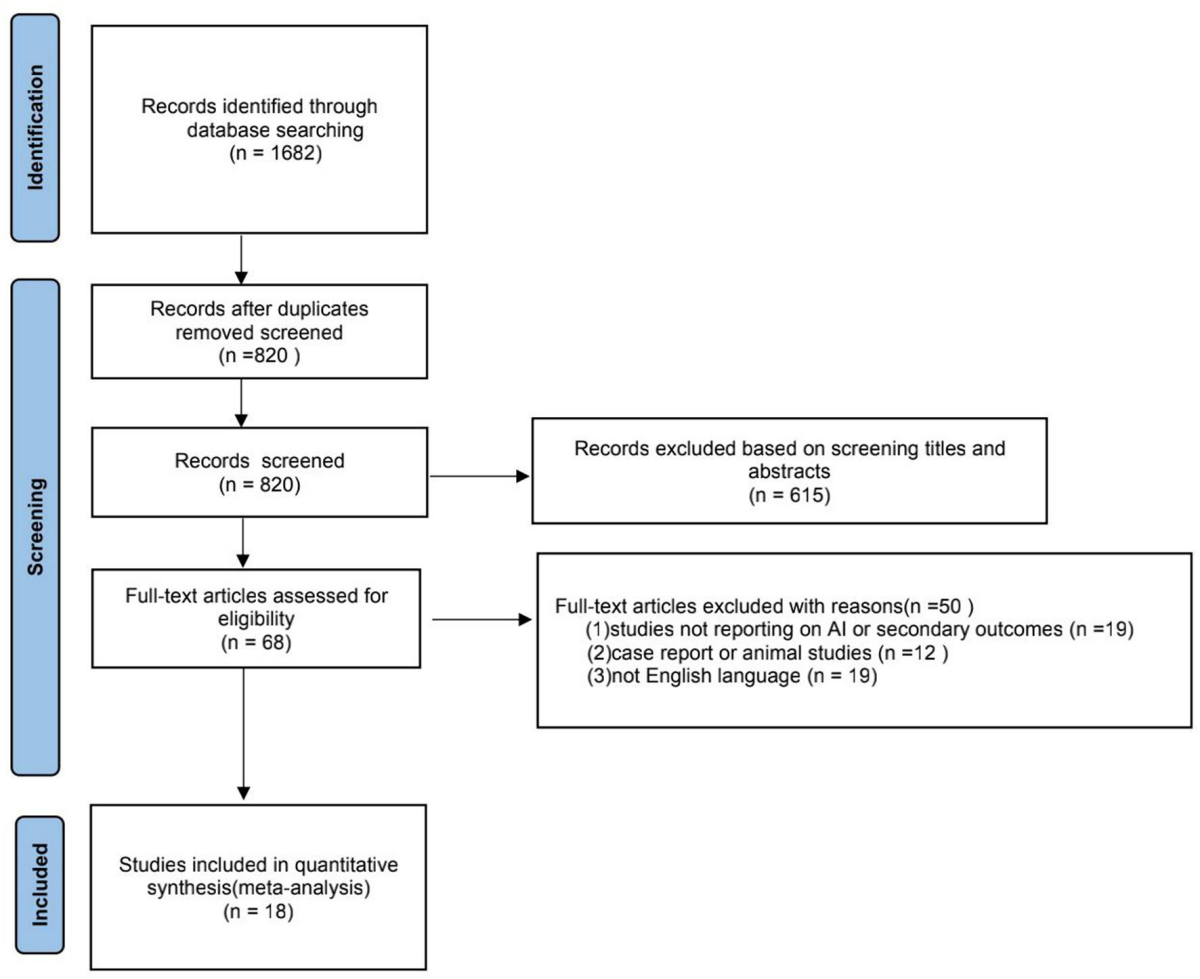
A flow chart of the selection process for the included studies.

We identified 1,682 records in accordance with the search strategy and assessed the full text of 68 studies for eligibility. A flow chart of the search process is presented in Figure 1. Of these 68 studies, 18 studies have satisfied all the inclusion criteria and were incorporated in the final data analysis (10–27). A total of 926 patients comprised 18 studies.

Table 1 presents the basic characteristics of the included trials and the number of participants. All the studies were published between 2008 and 2021. We identified 6 parallel-group RCTs (20, 22–25, 27) and 12 randomized crossover studies (10–19, 21, 26). The Jadad Scales of all the included studies ranged from 2 to 6, and the relatively low scores of the included studies resulted from the particularity of these studies that investigated the kinds of ventilation modes. The assessment of the risk of bias in the included studies is detailed in Supplementary Figures 1, 2. The overall quality of these studies was at a medium-to-low level. In these studies, the blind methods cannot be implemented because of the principle of study design, but it was applicable for outcomes evaluation. However, all the studies involved in our study were prospective, and RCTs are of higher quality in reducing selection bias.

**Table 1:** Baseline characteristics of these studies.

**References**	**Type**	**Jadad scale**	**Participants**	**Treat**	**Control**
Colombo et al. (10)	Randonmized, cross-over	1+2+0+1 = 4	14	NAVA	PSV
Schmidt et al. (11)	Randonmized, cross-over	1+1+0+0 = 2	12	NAVA	PSV
Piquilloud et al. (12)	Randonmized, cross-over	1+1+0+0 = 2	22	NAVA	PSV
Piquilloud et al. (13)	Randonmized, cross-over	1+1+0+0 = 2	13	NAVA	PSV
Bertrand et al. (14)	Randonmized, cross-over	1+1+2+0 = 4	13	NAVA	PSV
Vignaux et al. (15)	Randonmized, cross-over	1+1+0+0 = 2	19	NAVA	PSV
Doorduin et al. (16)	Randonmized, cross-over	2+1+1+1 = 5	12	NAVA	PSV
Baudin et al. (17)	Randonmized, cross-over	1+1+0+0 = 2	11	NAVA	PSV
Vignaux et al. (18)	Randonmized, cross-over	2+1+1+1 = 5	6	NAVA	PSV
Chidini et al. (19)	Randonmized, cross-over	2+1+1+1 = 5	18	NAVA	PSV
Demoule et al. (20)	RCT	1+2+0+1 = 4	128	NAVA	PSV
Ferreira et al. (21)	Randonmized, cross-over	2+2+1+0 = 5	20	NAVA	PSV
Kacmarek et al. (22)	RCT	2+2+1+1 = 6	306	NAVA	CMV
Hadfield et al. (23)	RCT	2+2+1+0 = 5	77	NAVA	PSV
Tajamul et al. (24)	RCT	2+1+1+1 = 5	40	NAVA	PSV
Liu et al. (25)	RCT	2+2+1+1 = 6	99	NAVA	PSV
Cammarota et al. (26)	Randonmized, cross-over	2+2+1+0 = 5	16	NAVA	PSV
Prasad et al. (27)	RCT	2+2+1+1 = 6	100	NAVA	PSV

*NAVA, neurally adjusted ventilatory assist; RCT, randomized controlled trial; PSV, pressure support ventilation; and CMV, conventional mechanical ventilation*.

### Primary Outcome

#### Patient-Ventilator Asynchrony Index

**Figure 2:**
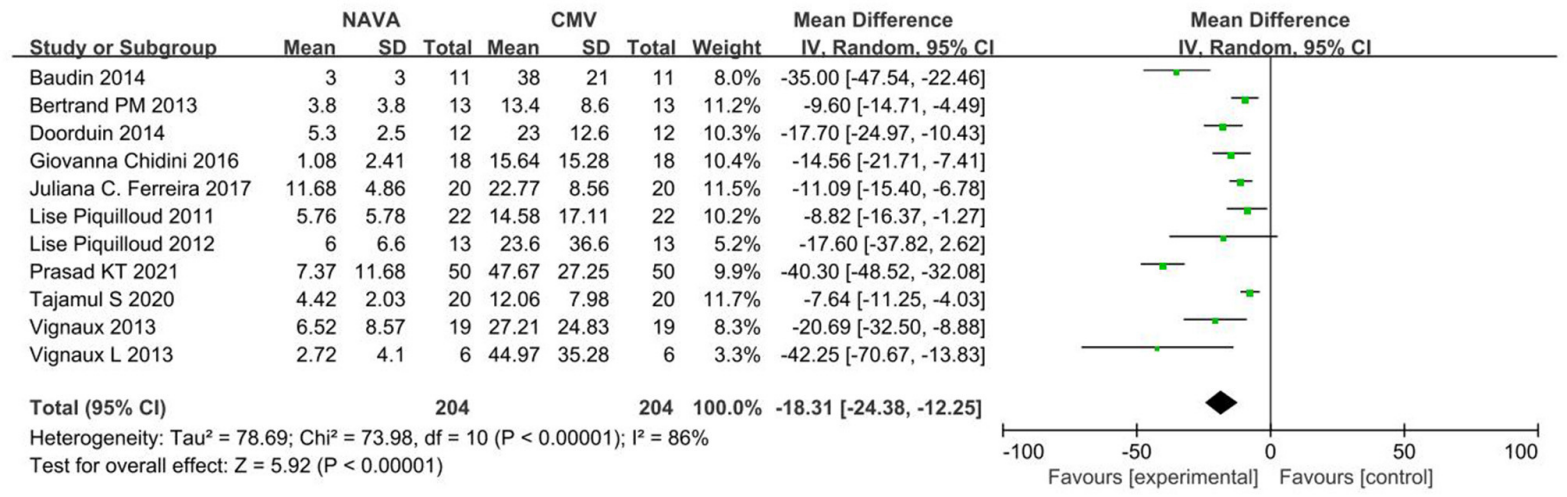
A forest plot for AI.

For the AI, our study included 11 studies (12–19, 21, 24, 27), with 274 patients in total. The AI was significantly lower in the NAVA group the than PSV group) (MD = −18.31; 95% CI, −24.38 to −12.25; *p* < 0.001; Figure 2). Heterogeneity testing showed that *I*^2^ = 89%, indicating a high heterogeneity. So, we used the random-effects model and subgroup analysis to solve it.

#### Subgroup Analysis

**Figure 3:**
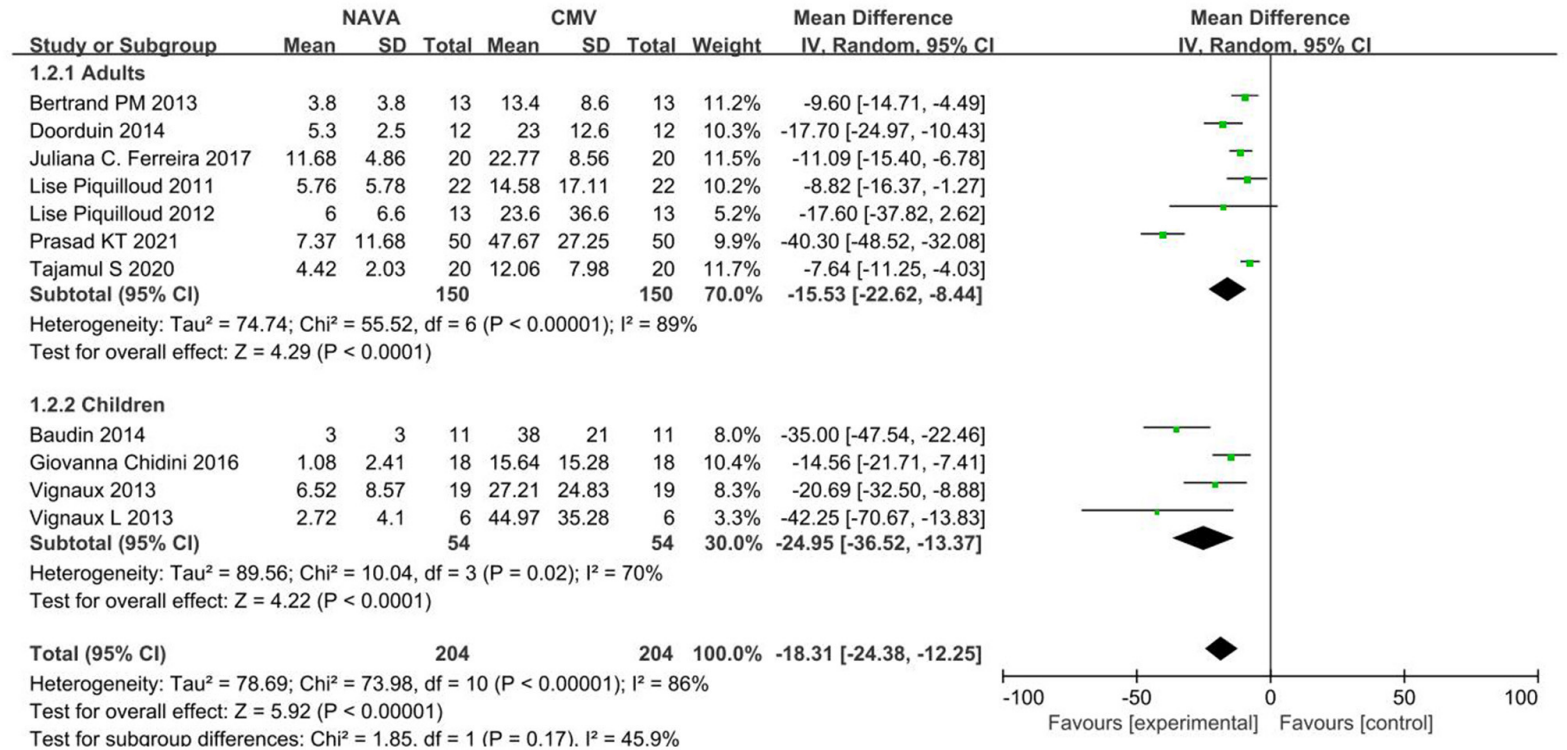
A forest plot for AI in adults and children.

**Figure 4:**
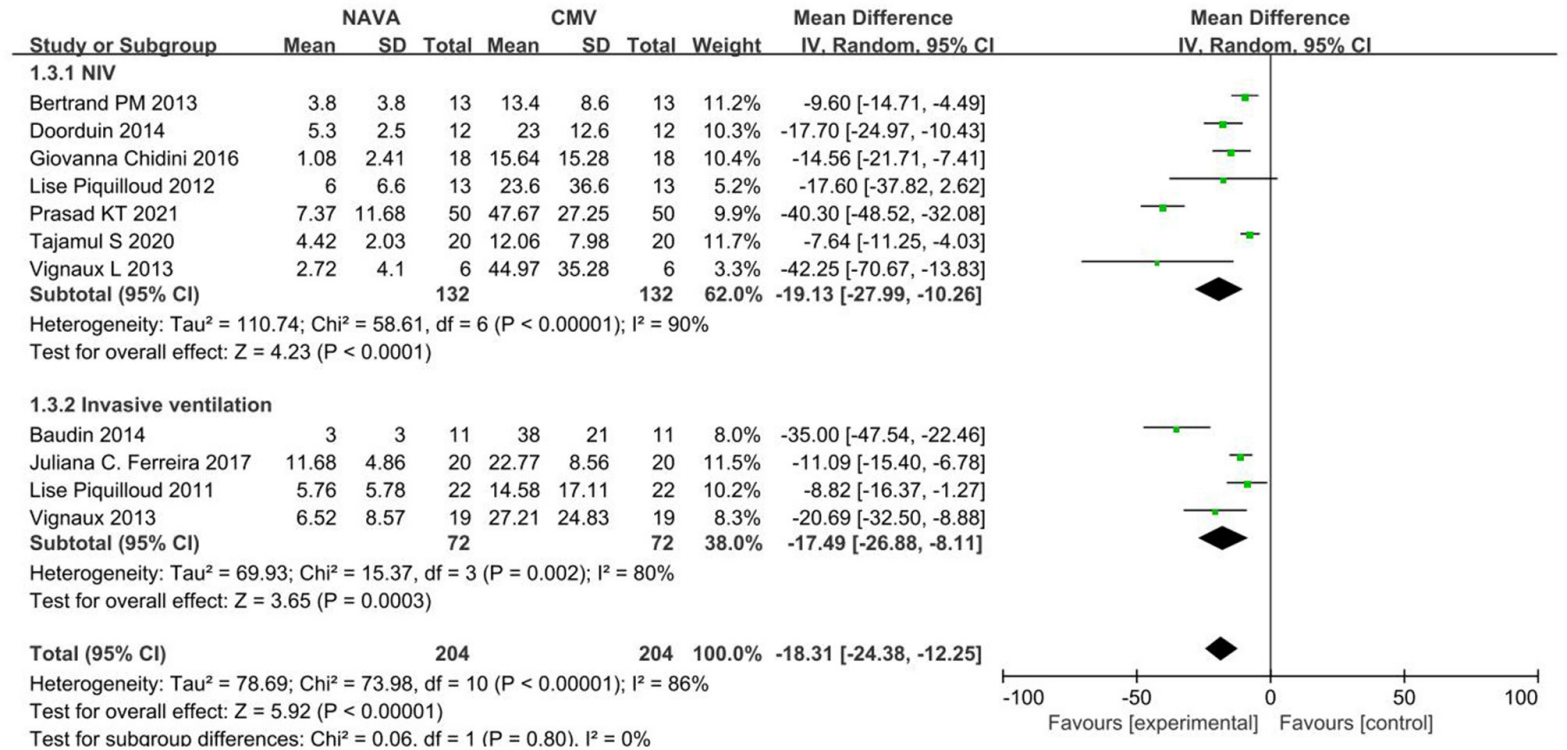
A forest plot for AI in NIV and invasive ventilation.

**Figure 5:**
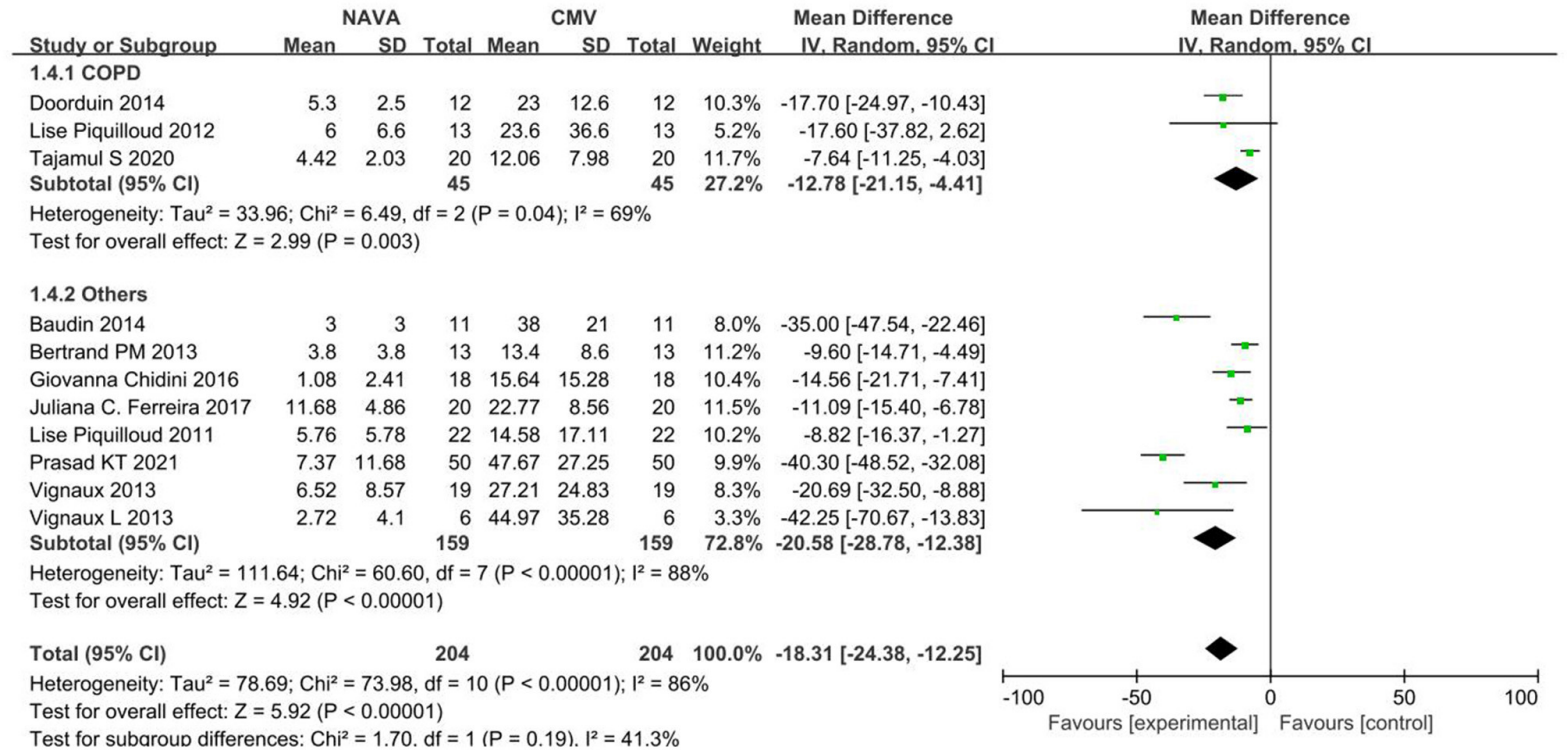
A forest plot for AI in patients with COPD or other causes.

Subgroup analysis grouped by age showed that the AI of NAVA was lower than the conventional MV modes in adults (MD, −15.53; 95% CI: −22.62 to −8.44; *I*^2^ = 89%), and children (MD, −24.95; 95% CI: −36.52 to −13.37; *I*^2^ = 86%; Figure 3). The AI of NAVA was lower in NIV (MD, −19.13; 95% CI: −27.99 to −10.26; *I* ^2^= 90%), and in invasive ventilation (MD, −17.49; 95% CI: −26.88 to −8.11; *I*^2^ = 80%; Figure 4). According to different causes of ARF, we divided studies into the COPD group and the others group. The AI of NAVA was lower compared with conventional MV modes in the COPD group (MD, −12.78; 95% CI: −21.15 to −4.41; *I*^2^ = 69%) and in the others group (MD, −20.58; 95% CI: −28.78 to −12.38; *I*^2^ = 88%; Figure 5).

A funnel plot on AI was evaluated and did not imply evidence of publication bias (Supplementary Figure 3). Sensitivity analyses showed that these studies might result in a high heterogeneity (Supplementary Figure 4). Noteworthy, meta-regression suggested that the year of publication, Jadad scale, and ventilation methods did not contribute to the high heterogeneity (Supplementary Figure 5).

### Secondary Outcomes

#### Duration of MV

**Figure 6:**
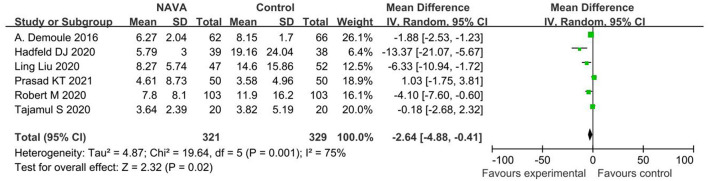
A forest plot for duration of MV.

For the result of ventilation days, our study included 6 studies (20, 22–25, 27), about a total of 650 patients, and showed that NAVA was significantly lower than other MV modes in ventilation days (MD = 2.64; 95% CI, −4.88 to −0.41; *p* = 0.02; Figure 6). Heterogeneity testing showed that *I*^2^ = 75%, indicating a high heterogeneity, so a random-effects model and a sensitivity analysis shown in Supplementary Figure 6 were used. The certainty of the evidence was moderate due to inconsistency.

#### ICU Mortality

**Figure 7:**
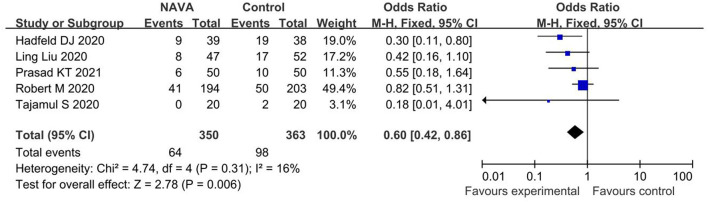
A forest plot for ICU mortality.

For the result of ICU mortality, our study included 5 studies (22–25, 27) with 713 patients in total, and the result proved that the ICU mortality of patients ventilated with NAVA was significantly lower than those of patients ventilated with conventional MV (OR,0.60; 95% CI, 0.42 to 0.86, *p* = 0.006; Figure 7). Heterogeneity testing showed that *I*^2^ = 16%, indicating a low heterogeneity.

#### Ventilator-Associated Pneumonia

**Figure 8:**
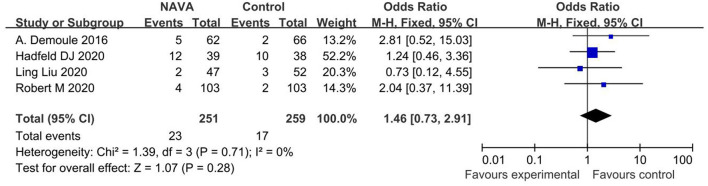
A forest plot for ventilator-associated pneumonia.

For the result of ventilator-associated pneumonia, our study included 4 studies (20, 22, 23, 25), with a total of 510 patients, and showed that there was no statistically significant difference in ventilator-associated pneumonia (OR, 1.46; 95% CI, 0.73 to 2.91, *p* = 0.006; Figure 8). Heterogeneity testing showed that *I*^2^ = 0%, indicating a low heterogeneity.

#### pH

**Figure 9:**
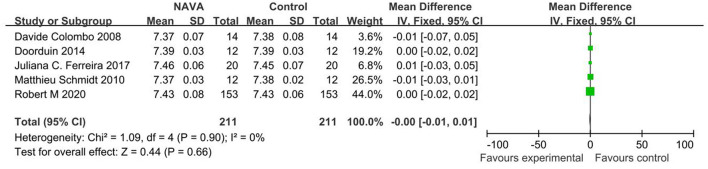
A forest plot for pH.

For the result of pH, our study included 5 studies (10, 11, 16, 21, 22), with 264 patients, and showed that there was no statistically significant difference between the NAVA group and the control group (MD = −0.00; 95% CI, −0.01 to 0.01; *p* = 0.90; Figure 9). Heterogeneity testing showed that *I*^2^= 0%, indicating a low heterogeneity.

#### PaCO2

**Figure 10:**
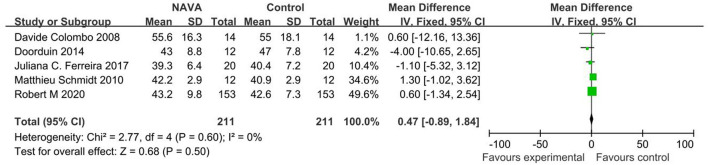
A forest plot for PaCO_2_.

For the result of PaCO2, our study included 5 studies (10, 11, 16, 21, 22), with 264 patients, and showed that there was no statistically significant difference between the NAVA group and the control group (MD =0.47; 95% CI, −0.89 to 1.84; *p* = 0.60; Figure 10). Heterogeneity testing showed that *I*^2^ = 0%, indicating a low heterogeneity.

## Discussion

Our systematic review and meta-analysis have identified 18 studies of 919 patients that evaluated the effect of NAVA on patient-ventilator interaction and clinical outcomes in patients with ARF compared with conventional MV modes. The key findings were that, compared with traditional modes of MV, NAVA has obvious advantages: (a) improving the patient-ventilator interaction; and (b) decreasing the duration of MV and ICU mortality. Subgroup analysis suggested that whether in adult patients or patients with the pediatric condition, invasive ventilation or NIV, COPD, or other causes, NAVA had the benefits in better patient-ventilator interaction. There are many other factors over and above the ventilation modes influencing the patient-ventilator interactions during NIV, such as the compliance and tolerance of the patient to the interface, different kinds of interface, psychological factors of patients, and so on. So, it is necessary to clarify the influence of NAVA on patient-ventilator interaction among many factors in further study.

Synchronization of patient-ventilator with MV has been the objective of numerous ventilation strategies. In this study, the significant decrease in AI in patients with NAVA can easily be explained by the fact that the EAdi, the temporal sum of the electromyographic potentials of the crural diaphragm recorded by means of a nasogastric tube, with multiple arrays of electrodes (5, 32), is used to trigger the ventilator rather than a pneumatic signal located at the airway opening or inside the ventilator (33, 34). The patients were ventilated with a ventilator equipped with the NAVA software that includes the “neuro-ventilatory tool” for EAdi measurement (35). After receiving these signals, the ventilator gives ventilation support according to the preset trigger range and the support level. The ventilation support pressure level (unit: cmH_2_O) is determined by the product of the preset support level (unit: cmH_2_O/μV) and EAdi (unit:μV). In theory, NAVA is in line with the physiological characteristics of respiration and can maximize the synchronization of patient-ventilator. If the EAdi signal is lost, this mode reverts to PSV. To a certain extent, NAVA avoids the situation of over-assistance under-assistance because the level of ventilation support is matched with the respiratory drive through feedback regulation of EAdi. Over-assistance would put the patient at risk of diaphragmatic atrophy, while, on the other hand, under-assistance would result in dyspnea, diaphragmatic fatigue, and patient self-inflicted lung injury. The Eadi, according to Bellani et al. (36), with adequate measurements, could be considered a surrogate of work of breathing. Optimizing the work of breathing may reduce the incidence and change the quality of asynchrony. It should be noted that double triggering was more frequent in NAVA than in CMV in our study, which followed the results of Piquilloud and colleagues (12). The reason for the prevalence of double-triggering during NAVA is the biphasic appearance of EAdi signals, which could be related to early cycling when the inspiratory time of the ventilator is less than the neural inspiratory time of the patient, and this causes two successive cycles. This may not increase the work of breathing, but it may participate in the discomfort felt by patients (12).

This study is the first to appraise the clinical prognosis in patients undergoing NAVA. A recent report of a review (37) has observed the association of NAVA with better patient-ventilator synchrony in comparison with PSV in mechanically-ventilated adults. However, its effects on clinical outcomes remain uncertain. Previous studies have shown that patient-ventilator asynchrony may lead to lung and vascular damage, resulting in adverse clinical outcomes, including a prolonged MV (38), increased mortality (39), intensive care unit and hospitalization (40), discomfort (41), and sleep disturbances (42). Our study found that NAVA was associated with a reduction in the duration of MV and ICU mortality. Some short-term physiologic crossover studies with small sample sizes (10, 12, 14) in our systematic review provided definite conclusions on the clinical effect of NAVA, but heterogeneous inclusion criteria, asynchrony detection methods, and NAVA titration strategies are still needed. Some studies (19, 30) reported that NAVA might further decrease the ICU mortality and the ventilator-associated pneumonia incidence when compared with PSV. Furthermore, it has been manifested that NAVA could improve the success rate of direct weaning from the ventilator (2, 42). These beneficial effects could be examined in multiple different clinical situations, such as the comfort degree of patients, depth of sedation, patients sustained with ECMO, and long-term respiratory rehabilitation. Considering that MV is related to complications, such as a ventilator-induced lung injury and a ventilator-induced diaphragmatic dysfunction, the physiologic benefits of NAVA are expected to improve the clinical outcomes (43).

Although this meta-analysis suggests that NAVA has advantages in improving physiological and important clinical outcomes in ARF patients with MV, notably, NAVA, still, has some potentially relevant boundedness such as the necessary condition for the application. The accurate positioning of the NAVA catheter is necessary (44). Nevertheless, the sensitivity of the electrode will be affected by many factors, such as the position and time of placement, depth of sedation, and muscle relaxants. Therefore, ventilation in reserve is required to ensure the safety of patients. The need for specific equipment and an intact neuromuscular transmission, the persistence of double triggering (16, 29, 45–47), and the occurrence of hypervariable respiratory patterns at high-assistance levels (34, 45) are also limitations.

Limitations of this study exist as well. First, the quantitative synthesis of some endpoints was only composed of four or five studies that were pooled so that there were not enough data to assess the incidence of ventilator-associated pneumonia or blood gas results (pH and PaCO2), which may explain why some of the experimental results are not consistent with the expected situation. On the other hand, no pediatric or neonatal study could, so far, show an impact on the outcome with the use of NAVA; thus, we can only focus on adults and children, and the results cannot be extended to the general population. Nonetheless, these were pooled to visually depict the pooled effect as well as to quantify the pooled effect. Second, some of the included studies are crossover trials, which is a theoretical risk that the efficacy of NAVA may be overestimated or underestimated compared with that of other CMV modes. Third, a relatively large number of studies on Europeans and Americans had been included. It may reduce the applicability of our results to different races. Another limitation is that they used the variable definitions of outcomes (e.g., duration of MV) in the included studies despite attempts to reduce the clinical heterogeneity. Finally, all studies in our analysis had a high risk of performance bias because of the inability to blind the investigators. So, it is possible that the decisions and actions of the investigators may be influenced, resulting in biased estimates of results.

In conclusion, NAVA ameliorates the patient-ventilator synchrony and improves the clinical outcomes of patients (especially in adults) with ARF compared with CMV modes. Although our research suggests that NAVA is beneficial in physiological and clinical outcomes, a large number of RCTs of neonates are still needed to verify its reliability.

## Data Availability Statement

The original contributions presented in the study are included in the article/Supplementary Material, further inquiries can be directed to the corresponding author/s.

## Author Contributions

MW and XY searched the scientific literature and collected the data. MW drafted the manuscript and performed statistical analyses. YY contributed to the conception, design, data interpretation, manuscript revision for critical intellectual content, and supervision of the study. LL participated in data interpretation and revision of the manuscript. All authors have read and approved the manuscript.

## Funding

This work was supported by the National Natural Science Foundation of China (Grant No. 81870066) and Clinical Science and Technology Specific Projects of Jiangsu Province (BE2020786, BE2019749).

## Conflict of Interest

The authors declare that the research was conducted in the absence of any commercial or financial relationships that could be construed as a potential conflict of interest.

## Publisher's Note

All claims expressed in this article are solely those of the authors and do not necessarily represent those of their affiliated organizations, or those of the publisher, the editors and the reviewers. Any product that may be evaluated in this article, or claim that may be made by its manufacturer, is not guaranteed or endorsed by the publisher.
