# Supplementary material for: Neurally Adjusted Ventilatory Assist vs. Conventional Mechanical Ventilation in Adults and Children With Acute Respiratory Failure: A Systematic Review and Meta-Analysis
Source: Front Med (Lausanne). 2022 Feb 22;9:814245. doi: 10.3389/fmed.2022.814245 (PMC8901502; doi:10.3389/fmed.2022.814245)
Supplement: Supplementary file 1 [file Data_Sheet_1.docx]

**Additional file 1**

**Figures:**


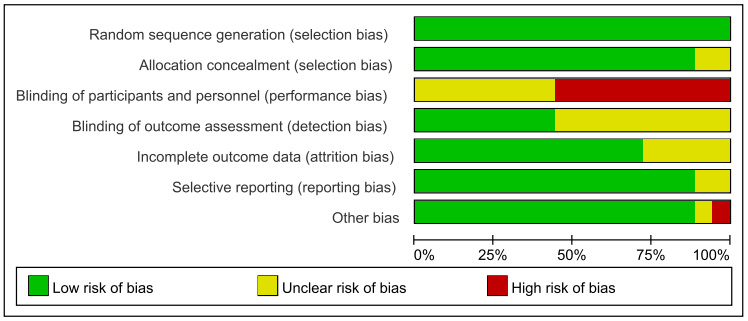


**Supplementary Figure 1.** Risk of bias graph


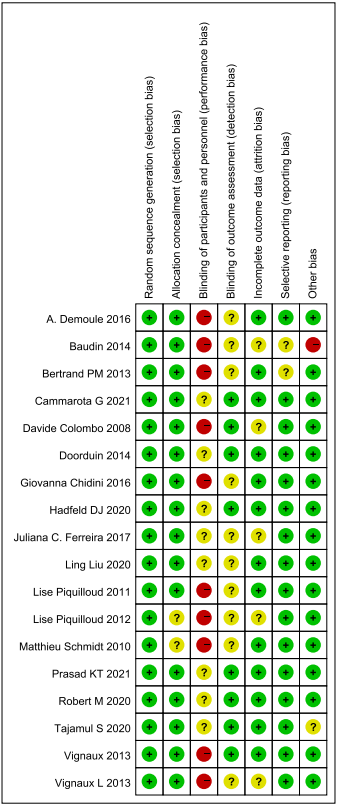


**Supplementary Figure 2.** Risk of bias summary


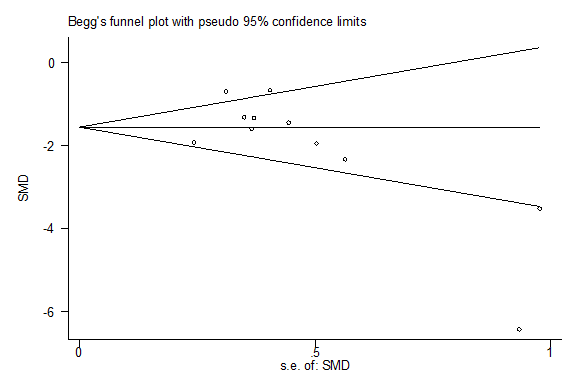


**Supplementary Figure 3.** Funnel plot of publication bias

**Supplementary Figure 4.** Sensitivity analyses for AI


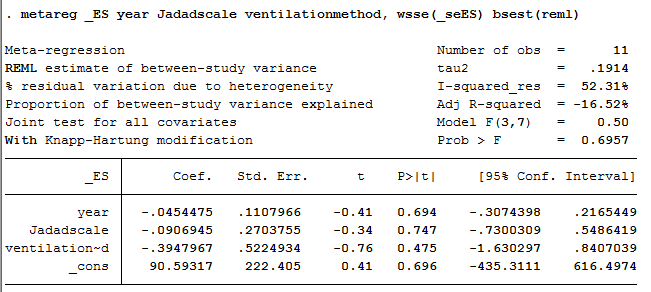


**Supplementary Figure 5.** Meta regression for AI

**Supplementary Figure 6.** Sensitivity analyses for duration of MV
